# Alpha-Lipoic Acid: Biological Mechanisms and Health Benefits

**DOI:** 10.3390/antiox13101228

**Published:** 2024-10-12

**Authors:** Fabiana Superti, Rosario Russo

**Affiliations:** 1 Istituto Superiore di Sanità, Viale Regina Elena, 299, 00161 Rome, RM, Italy; fabiana.superti@iss.it; 2 Association for Research on Integrative Oncology Therapies, (ARTOI) Foundation, Via Ludovico Micara, 73, 00165 Rome, RM, Italy; 3 Giellepi S.p.A., Via G. Verdi, 41/Q, 20831 Seregno, MB, Italy

**Keywords:** alpha-lipoic acid, antioxidant activity, anti-inflammatory activity, food supplement, neuroprotection

## Abstract

Alpha-lipoic acid (ALA) is a bioactive molecule with significant health effects. The biological action of ALA has been ascribed to the characteristic antioxidant properties of the oxidized form (ALA) and its reduced counterpart the dihydrolipoic acid (DHLA) system. The ALA/DHLA combination represents an ideal antioxidant since it can quench radicals, is able to chelate metals, is amphiphilic, and has no major adverse effects. This unique system is able to scavenge reactive oxygen species, exerting a major effect on tissue levels of reduced forms of other antioxidants, including glutathione. For this reason, ALA is also known as the “antioxidant of antioxidants”. This review analyzes the antioxidant, anti-inflammatory, and neuroprotective effects of ALA and discusses its applications as an ameliorative tool for chronic diseases and those associated with oxidative stress. Results from in vitro and in vivo studies demonstrated that ALA modulates various oxidative stress pathways suggesting its application, alone or in combination with other functional substances, as a useful support in numerous conditions, in which the balance oxidant–antioxidant is disrupted, such as neurodegenerative disorders. Based on several successful clinical studies, it has been also established that oral ALA supplements are clinically useful in relieving the complications of diabetes and other disorders including cardiovascular diseases and nerve discomforts suggesting that ALA can be considered a useful approach to improving our health.

## 1. Introduction

Alpha-lipoic acid (ALA), IUPAC name: 5-(1,2-dithiolan-3-yl) pentanoic acid, is a natural compound endowing different beneficial effects for health.

ALA, also known as thioctic acid or dithioctanoic acid, is an endogenous disulfide derivative of octanoic acid produced in mitochondria which was first isolated by Reed and co-workers in 1951 [1]. In addition to playing a crucial role in mitochondrial energy metabolism, ALA, which is easily absorbed from the gut and crosses the blood–brain barrier, is known for its potent antioxidative effects [2]. ALA also exerts an indirect antioxidant action, favoring the action of other antioxidant substances (such as vitamin E, vitamin C, and coenzyme Q10). Due to these important properties, it is used as a dietary supplement and therapeutic for the treatment of chronic diseases linked to high levels of oxidative stress.

In nature, the richest sources of ALA are animal tissues with high metabolic activity such as the heart, liver, and kidney [3]. Among the vegetable sources, the highest ALA content is found in spinach followed by broccoli, tomatoes, peas, Brussels sprouts, and rice [4,5]. However, dietary absorption from these sources is considered insufficient, not delivering substantial amounts of ALA into the bloodstream [6].

**Figure 1:**
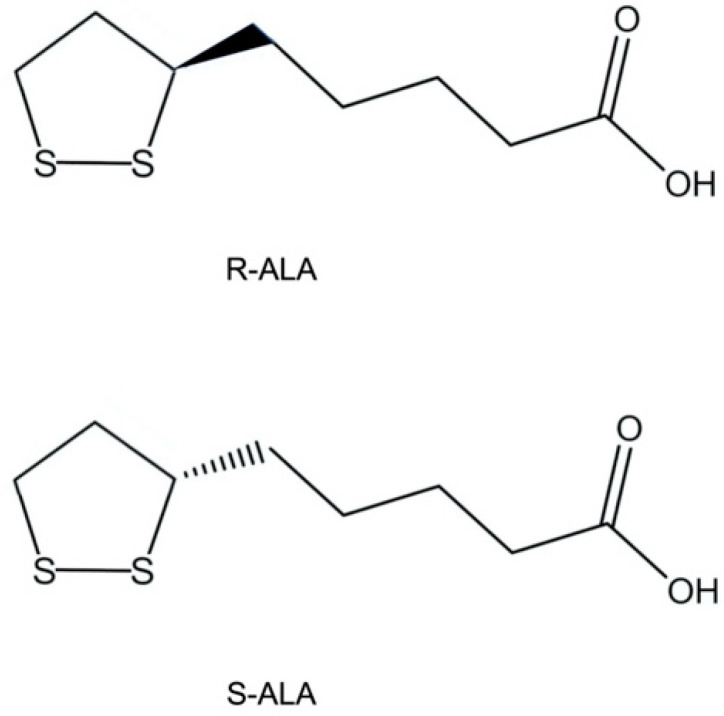
R and S enantiomers of alpha-lipoic acid.

ALA contains a chiral center and, thus, exists in two enantiomeric forms: R-ALA and S-ALA. In nature, ALA is found only in the R form (Figure 1) and is present in the body both in free form and conjugated to conserved lysine residues in an amide bond, thus making this isomer a key cofactor in different biological systems [7].

Due to its amphiphilic structure, ALA is soluble in both aqueous and lipidic environments. This extremely advantageous feature allows its distribution throughout the body, including the central nervous system as it can cross the blood–brain barrier [6,8].

**Figure 2:**
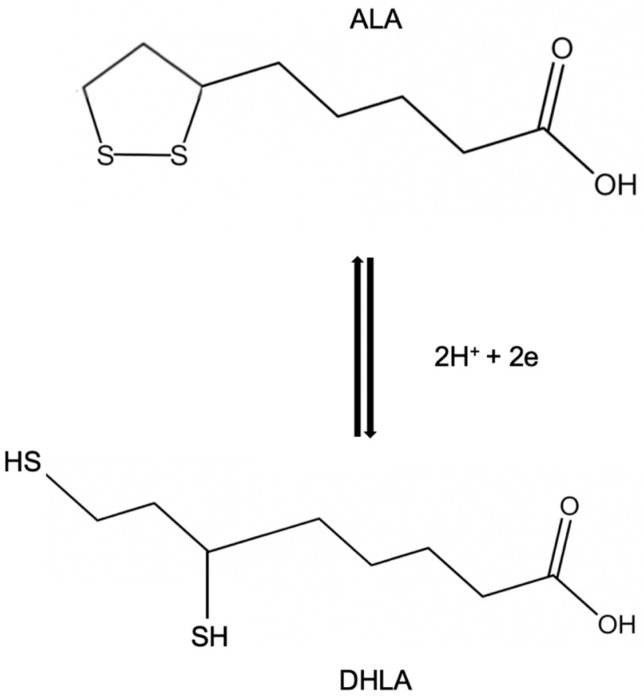
Chemical structure of alpha-lipoic acid (ALA) and its reduced form dihydrolipoic acid (DHLA).

In vivo ALA can be reduced to the dithiol form (where the ring structure is broken) called dihydrolipoic acid (DHLA). The oxidized form ALA and its active reduced counterpart DHLA (Figure 2), two of the major powerful antioxidants present in nature, have been demonstrated to fight oxidative stress by quenching a variety of reactive oxygen species (ROS) [9].

## 2. Alpha-Lipoic Acid as an Antioxidant

Among antioxidant substances, alpha-lipoic acid is unique as it maintains protective functions in both its oxidized (ALA) and reduced (DHLA) forms (Table 1) [8,10], although the latter is more effective in performing antioxidant functions. ALA and DHLA forms create a potent redox couple that has a standard reduction potential of −0.32 V. In particular, DHLA is able to regenerate other endogenous antioxidants (for example, vitamins E and C) [11] and neutralize free radicals. Both ALA and DHLA have the ability to prevent the formation of protein carbonyl by scavenging hypochlorite [12]; they can scavenge hydroxyl radicals and hypochlorous acid [6] while neither form is active against hydrogen peroxide at significant rates [13]. ALA and DHLA, having a relatively low molecular mass, are soluble in both water and lipid environments; therefore, they connect the activity of antioxidants in the membranes and in the cytoplasm, increasing the antioxidant network inside the cells.

It is well known that it is necessary to examine different parameters to evaluate the antioxidant capacity of a compound such as the interaction with other antioxidant molecules, the specificity in the elimination of free radicals, the metal-chelating ability, the outcomes on gene expression, the capability to repair oxidative injury, and the bioavailability [10]. The ALA/DHLA redox couple, fully satisfying these parameters, can be defined as an excellent antioxidant. As stated previously, the antioxidant potential of ALA is lower than DHLA, which is capable of inhibiting lipid peroxidation [8]. The latter represents an important aspect of oxidative damage as it leads to alteration of membrane structure and barrier function [14]. The antioxidant efficiency of ALA and DHLA was tested by studying their interactions with different types of radicals, and DHLA was shown to be effective in scavenging activity directed toward peroxyl radicals both in the membrane and in the aqueous phase without any need for glutathione (GSH) or α-tocopherol to carry out its antioxidant activity [8]. DHLA has also been shown to reduce ascorbyl radicals produced during the oxidation of ascorbate by tocopheroxyl radicals [8]. Regarding singlet oxygen, it reacts only with ALA to form thiosulfinates and thiosulfonates [14].

Concerning the metal-chelating ability, both DHLA and ALA, due to the presence of nearby sulfur atoms and a carboxyl group, are capable of chelating redox-active metals. The activity of ALA/DHLA as chelating molecules does not result in metal depletion. In vitro studies showed that DHLA in addition to Co^2+^, Ni^2+^, Cd^2+^, Cu^2+^, Zn^2+^, Pb^2+^, and Hg^2+^ also chelates Fe^3+^ (its complex is more stable than that formed with Fe^2+^), whereas ALA preferentially binds Mn^2+^, Cu^2+,^ Zn^2+^, and Pb^2+^ but cannot chelate Fe^3+^ [14,15]. ALA is able to form a lipophilic complex with Cu^2+^ and protect against Cu^2+^-induced lipid peroxidation; by the same mechanism, DHLA prevents Cu^2+^- and Cd^2+^-induced lipid peroxidation [16]. It has been demonstrated that the chelation of Cu^2+^ and Fe^3+^ by DHLA in the brain reduces free radical damage and may exert a protective action in the pathobiology of Alzheimer’s disease [17].

**Table 1:** Antioxidant effect of ALA and/or DHLA on ROS.

^1^ Reactive Species (Oxidant)	ALA/DHLA	References
H_2_O_2_	ALA/DHLA	[5]
	DHLA	[13,18]
HO_2_	ALA/DHLA	[13]
	DHLA	[19]
O_2_^•^	DHLA	[5,18,19,20]
^•^HO	ALA/DHLA	[20]
	ALA	[13]
^1^O_2_	ALA/DHLA	[21,22]
	ALA	[5,23]
^•^NO	ALA/DHLA	[5]
ONOO^−^	ALA/DHLA	[5]
HOCl	ALA/DHLA	[13,24]
	ALA	[10]

^1^ H_2_O_2_, hydrogen peroxide; HO_2_, hydroperoxy radical; O_2_^•^, superoxide radical; ^•^HO, hydroxyl radical; ^1^O_2_, singlet oxygen; ^•^NO, nitric oxide radical; ONOO^−^, peroxynitrite; HOCl, hypochlorous acid.

**Figure 3:**
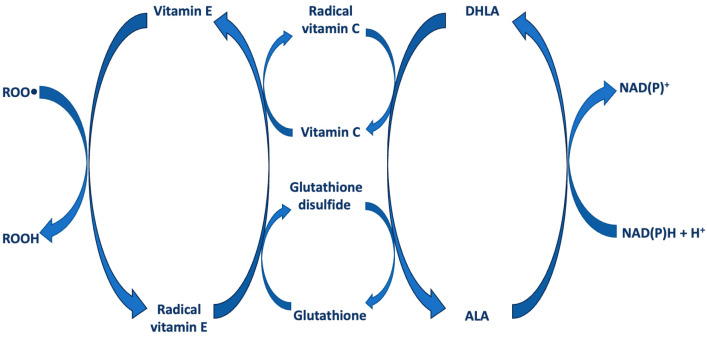
Role of ALA and DHLA in the regeneration cycle of cellular antioxidants (ROO^•^ = peroxyl radical; ROOH = hydroperoxide; NAD = nicotinamide adenine dinucleotide; NADP = nicotinamide adenine dinucleotide phosphate; NAD(P)H = reduced NAD or NADP).

ALA/DHLA can also maintain cellular antioxidant status indirectly by inducing the absorption of other antioxidant molecules or enhancing the synthesis of endogenous antioxidants, such as vitamin C (ascorbic acid) and, indirectly, vitamin E, or antioxidant enzymes and the ratio of reduced/oxidized glutathione (GSH/GSSG) [25] (Figure 3). Vitamin C is a water-soluble vitamin that acts as an antioxidant, protecting against damage by reactive free radicals [26]. Vitamin E is a group of eight lipid-soluble compounds: four tocopherols and four tocotrienols [27].

As early as 1959, Rosenberg and Culik [28] suggested the protection of other antioxidants by ALA demonstrating that ALA is able to prevent symptoms of both vitamin E and vitamin C deficiency. In particular, they stated “*α-Lipoic acid, and even more so its dihydro derivative to which it is rapidly converted after entering cellular metabolism, could act as an antioxidant for ascorbic acid and tocopherols*”.

ALA increased also the level of GSH both in in vitro and in vivo experimental models [29,30]. GSH is a natural tripeptide, made up of glutamic acid, cysteine, and glycine. This particular chemical composition gives GSH, an important endogenous antioxidant, a high capacity to oxidize or reduce, protecting proteins and other oxidizable compounds from the toxic action of free radicals. ALA enhances and maintains cellular levels of GSH by acting as a transcriptional inducer of the genes that regulate its synthesis and by increasing substrate availability [31].

Thus, ALA and DHLA act as antioxidants not only directly, through metal chelation and free radical quenching, but also indirectly, both through the recycling of other antioxidants and by inducing an increase in intracellular GSH levels.

## 3. Anti-Inflammatory Activity of Alpha-Lipoic Acid

Inflammation is the consequence of an innate biological response to injurious agents. It represents a defense of the body that tries to remove harmful stimuli, protect the surrounding tissue, and start healing. Inflammation, if it becomes chronic, can lead to the development of different types of diseases. ALA has been shown to possess inhibitory properties toward nuclear factor kappa B (NF-kB) [32,33,34], an important proinflammatory transcription factor that regulates numerous genes involved in inflammation, such as those encoding adhesion molecules, monocyte chemotactic protein-1, and several cytokines. As a matter of fact, ALA is able to prevent the upregulation of intercellular adhesion-molecule-1 (ICAM-1) and vascular cell adhesion-molecule-1 (VCAM-1) in tumor necrosis factor α (TNF-α)-stimulated brain and aortic endothelial cells in vitro [33,35]. It is important to underline that although ALA has been investigated in cytokine-induced inflammation for its antioxidant characteristics (enhanced oxidative stress is essential for persistent inflammation), its inhibitory effects on TNF-α-induced endothelial activation are thought to be due to its metal chelating activity rather than a general antioxidant effect. Concerning the inhibition of NF-kB activation, it occurs through the inhibition of degradation of its cytosolic dock IkB (inhibitor of NF-kB). When NF-kB interacts with IkB in the cytoplasm, it is unable to enter the nucleus and bind to the promotor region of proinflammatory genes.

## 4. Alpha-Lipoic Acid as a Neuroprotective Agent

A primary lesion or malfunction resulting in damage to the peripheral nerve system is referred to as peripheral neuropathy (PN). PN is classified as either mononeuropathy (affecting one nerve) or polyneuropathy (involving several nerves). The two most prevalent mononeuropathies are Bell’s palsy, which affects the facial nerve and results in facial paralysis, and carpal tunnel syndrome (CTS), which is mostly due to compression and mechanical damage to the median nerve leading to ischemia and locally resulting in demyelination of the median nerve [36]. Diabetes, thyroid dysfunctions, vitamin B12 insufficiency, alcoholism, chemotherapy, and human immunodeficiency virus (HIV) infection are the most frequent causes of polyneuropathy. In a third of PN cases, despite in-depth analyses, no etiology is found; these cases are classified as idiopathic. The European Federation of Neurological Societies guidelines on neuropathy recommend pregabalin, gabapentin, duloxetine, tricyclic antidepressants, and venlafaxine for first-line treatment, with opioids and tramadol reserved for second-line therapy [37]. Neuropathy, in particular PN, has no FDA-approved disease improvement therapy. The goal of PN clinical treatment is to relieve metabolic problems, repair nerves, reduce oxidative stress, and improve microcirculation and other clinical symptoms. Hence, there is a demand for scientifically supported nutraceutical substances that maintain nerve health.

The brain consumes more oxygen than other organs, has more polyunsaturated fatty acids, and contains little catalase activity [38]. Superoxide dismutase (SOD) is localized primarily in neurons [39], whereas GSH peroxidase is localized primarily in astrocytes [40,41]. These enzyme localizations mean that neuronal tissue may be particularly vulnerable to H_2_O_2_, resulting in the formation of ROS that are known to be involved in a number of acute and chronic pathological conditions in the brain and neural tissue. In particular, depletion of GSH in disorders, such as cerebral ischemia–reperfusion [42] and Parkinson’s disease [43], leads to significant neurological damage [44,45].

Both ALA and DHLA are ideal substances to treat brain and neural oxidative disorders involving free radical processes because, as previously mentioned, they are potent antioxidants that act in the intracellular and extracellular milieu, regenerate other antioxidants such as vitamins C and E, and increase intracellular GSH levels [46]. Indeed ALA, absorbed from the diet and being able (as well as DHLA) to cross the blood–brain barrier [16,47], is used for relieving the complications of many neurological disorders such as diabetic polyneuropathy and multiple sclerosis. Furthermore, DHLA increases the activity of choline acetyltransferase, an enzyme essential for cognitive functions and neuronal homeostasis [48]. Regarding the inflammatory component in neuronal disorders, ALA and DHLA inhibit inflammasomes in neural tissues by decreasing pro-inflammatory mediators, such as interleukin-2 (IL-2), IFN-γ, and TNF-α, increasing anti-inflammatory cytokines such as interleukin-10 (IL-10), and regulating the transcription of nuclear factors Nrf2 and NF-κB in physiological processes. Therefore, ALA and DHLA, acting on different mechanisms involved in the pathogenesis of neurodegenerative disorders, can be considered “broad-spectrum” neuroprotective agents [47].

## 5. Pharmacokinetics of Alpha-Lipoic Acid

ALA is mainly taken as a dietary supplement by oral route. Upon ingestion, ALA is absorbed primarily in the small intestine, and peak plasma concentrations (C_max) are reached within 30 to 60 min, indicating rapid absorption [49]. Food intake may affect bioavailability, as consuming ALA with a meal can significantly reduce its absorption, likely due to delayed gastric emptying and interactions with dietary components [49]. Moreover, the low pH of the empty stomach promotes the absorption of ALA through the gastric mucosa [50].

Following absorption, ALA demonstrates efficient tissue targeting. It is transported in the bloodstream primarily bound to albumin [51]. Its amphipathic nature allows it to cross cellular membranes efficiently, facilitating entry into a variety of tissues. ALA demonstrates a particular affinity for the liver, heart, and skeletal muscle. Importantly, ALA can cross the blood–brain barrier, reaching the central nervous system, which is essential for its potential neuroprotective effects [47].

Mignini and co-workers showed that racemic ALA formulations (600 mg) have rapid absorption, short half-life, and low toxicity [52]. Moreover, the same research group [53] examined the bioavailability and pharmacokinetics of various ALA formulations after oral ingestion by healthy participants. The study carefully assessed the softgel and tablet formulations and concluded that 600 mg racemic ALA (BETTERAL^®^) exhibits a great safety profile in both dose forms along with rapid absorption and good bioavailability. These results highlighted the importance of a formulation that enables the molecule to elicit antioxidant activity at the cellular level at the highest concentrations and in the shortest amount of time. Finally, Amenta et al. [54] previously conducted another pharmacokinetic study on several ALA-based food supplements available on the Italian market obtaining similar results.

## 6. Alpha-Lipoic Acid as a Food Supplement

ALA is present in the global nutritional supplement market under different oral formulations such as film-coated tablets (generally 600 mg), soft capsules (300–600 mg), hard capsules (200 mg), powder, and aqueous liquids [55].

ALA marketed as a food supplement, when not specified, is a racemic solution of both the S and R isomers, while R-ALA, the naturally occurring form of biologically active ALA, is commonly bound to sodium (Na-R-ALA) as sodium salt. It has not yet been clearly established which of R-ALA, Na-R-ALA, or the racemic solution is the most suitable form for its use. It has been hypothesized that R-ALA would be the most appropriate form of commercial supplement; however, it is prone to polymerization and is absorbed to an even lower extent than racemic-ALA. As a matter of fact, it has been demonstrated that the presence of the S-enantiomer in the racemic mixture prevents the polymerization of R-ALA to some extent, increasing its bioavailability [6]. For this reason, the best nutraceuticals or food supplements available on the market are represented by highly pure forms of food-grade racemic ALA in order to keep the polymer content at a low level ensuring stability over time and optimal absorption. These products are often available in various forms such as fine powder for softgel formulations or ready-to-use granules for tablet preparations.

## 7. Alpha-Lipoic Acid Applications

It is known that ALA affects carbohydrate and lipid metabolism [56,57], regulates appetite [58], fights complications of diabetic origin [59] and myocardial and cerebral reperfusion injuries [60], supports the therapy of neurodegenerative diseases [61], and activates apoptosis in tumor cell lines [62]. So, we can state that ALA triggers pleiotropic effects due to its different and numerous mechanisms of action. The main applications of ALA are described below.

### 7.1. Alpha-Lipoic Acid for Treatment of Diabetes and Diabetes-Related Chronic Complications

Diabetes is a chronic disorder that develops when the pancreas does not produce sufficient insulin (type 1) or when the body is unable to use the insulin it produces (type 2). Since insulin is a hormone that regulates blood sugar, hyperglycemia is a consequence of uncontrolled diabetes, which can induce significant damage to several body systems, especially nerves and blood vessels. Type 2 diabetes is the most common form of diabetes accounting for about 90% of cases of this disease, and oxidative stress is believed to be a pivotal factor in its development [63]. So, treatment with ALA is indicated for prediabetics, diabetics, and diabetic complications.

The chronic complications of type 2 diabetes affect several organs and tissues, including the eyes, kidneys, heart, blood vessels, and peripheral nerves. These are diabetic retinopathy, diabetic nephropathy, cardiovascular disease, diabetic neuropathy, and pregnancy complications.

#### 7.1.1. Preclinical Studies in Diabetic Experimental Models

Concerning diabetic retinopathy, in vivo studies in diabetic rats demonstrated that long-term administration of ALA exerts inhibitory effects on the development of diabetic retinopathy through inhibition of the accumulation of oxidatively modified DNA, indicating that ALA supplementation as adjunctive therapy may help prevent vision damage in patients with diabetes [64].

Regarding diabetic nephropathy, results of studies on diabetic rats showed that ALA exerts a strong protective effect on diabetic kidneys [65] and that oral ALA supplementation is able to prevent early development and progression of diabetic nephropathy acting as an anti-hyperglycemic, antioxidant, anti-inflammatory, anti-fibrotic and anti-apoptotic compound [66].

In relation to diabetic cardiovascular diseases, in vivo studies in diabetic rats showed positive effects of ALA on the aortic arterial wall and angiogenic factors in the cardiovascular system, mainly on the myocardium [67,68].

As regards diabetic neuropathy, up to 50% of patients with diabetes have diabetic peripheral neuropathy (DPN), which is a significant contributor to morbidity and higher death rates. Its clinical signs include insensitivity and excruciating neuropathic pain, which raises the possibility of burns, wounds, and foot ulcers. A number of recent studies have linked the development of DPN to poor glycemic management, diabetes duration, hyperlipidemia (especially hypertriglyceridemia), higher albumin excretion rates, and obesity. There is currently little research on dietary components alone or in combination to alleviate common symptoms of poor nerve health. Experimental data, however, suggest that ALA supplementation may be beneficial for people who experience pain, discomfort, or nerve pain. As a matter of fact, preclinical studies in murine models showed that ALA and DHLA reduce neuronal damage, scavenging free radicals, regulating nerve growth factors (NGFs), decreasing lipid peroxidation, and hindering T lymphocytes and monocytes from migrating into the central nervous system [69,70]. Moreover, results of studies on rats with diabetic peripheral neuropathy demonstrated that ALA is able to alleviate peripheral neuropathy and improve peripheral nerve function (motor and sensory nerve conduction velocity). ALA reduces oxidative stress by activating the nuclear factor erythroid-2-related factor 2 and reduces neuronal apoptosis [71].

#### 7.1.2. Clinical Studies on Diabetes-Related Chronic Complications

Diabetes is associated with oxidative stress and lipid peroxidation of nerve membranes plays a key role in neuropathy.

A study conducted by Androne et al. [72] analyzed the effect of ALA on lipid peroxidation in patients with diabetic neuropathy. In this study, to investigate the extent of oxidative stress, serum ceruloplasmin (Cp) and lipid peroxide (Lp) levels were measured in 10 patients with diabetic neuropathy, before and 70 days after treatment with ALA (600 mg/day), and in 12 healthy control subjects, matched for age and sex. It was observed that serum Lp levels after ALA administration were significantly lower (*p* < 0.005) than those before treatment. This study demonstrated that antioxidant management with ALA improves and can prevent diabetic neuropathy and that this improvement is associated with a reduction in lipid peroxidation indices.

Tankova and co-workers [73] performed an open-label, randomized, controlled study to evaluate the effect of ALA on diabetic autonomic neuropathy. Seventy-five patients with type 1 diabetes and various forms of autonomic neuropathy were enrolled in this study: 46 patients (mean age of 38.1 ± 12.5 years) were treated with intravenous ALA (600 mg/day) for 10 days, then with oral ALA (600 mg film tablet/day) for 50 days, while 29 patients (mean age 40.2 ± 9.3 years) served as the untreated control group. A significant improvement in the score relating to the severity of cardiovascular autonomic neuropathy (CAN) (from moderate neuropathy: 6.43 ± 0.9 to mild neuropathy: 4.24 ± 1.8) was observed only in the treated patients. Regarding the laboratory parameters of oxidative stress, an increase in the serum antioxidant capacity was detected only in the treated group. These results demonstrate that ALA is an effective substance in the treatment of diabetic autonomic neuropathy.

The control of cardiovascular risk factors in patients with type 2 diabetes mellitus is essential for improving insulin sensitivity. The effect of oral administration of ALA on insulin sensitivity in these patients was evaluated [74]. In this study, oral ALA (600 mg twice daily) was administered for 4 weeks to 12 patients (mean age 52.9 ± 9.9 years). Twelve subjects with normal glucose tolerance were enrolled as a control group. The results of the study demonstrated that short-term oral treatment with ALA significantly increases peripheral insulin sensitivity in patients with type 2 diabetes mellitus, bringing it to a level almost similar to that of subjects in the control group.

The benefits of ALA in the management of diabetic peripheral and cardiac autonomic neuropathy have been reported by Ziegler and Gries [75]. In particular, two multicenter, double-blind, randomized, placebo-controlled studies have been described. The first study (ALA in Diabetic Neuropathy Study) enrolled 328 patients with symptomatic peripheral neuropathy and non-insulin-dependent diabetes mellitus who were randomized to receive an intravenous infusion of ALA at three different doses (1200 mg, 600 mg, or 100 mg/day) or a placebo for a period of 3 weeks. The results demonstrated that a 3-week intravenous course of ALA (1200 or 600 mg/day) was a safe and effective way to manage the symptoms of diabetic peripheral neuropathy. In the other trial (Deutsche Kardiale Autonome Neuropathie Studie), 73 patients with non-insulin-dependent diabetes mellitus and cardiac autonomic neuropathy were randomly assigned to the treated group (n = 39; ALA per os, 800 mg/day) or to the control group (n = 34; placebo) for 4 months. The results of the study demonstrated that oral treatment with ALA (800 mg/day) for 4 months can improve cardiac autonomic dysfunction in patients with non-insulin-dependent diabetes mellitus. No noteworthy adverse effects were noted in both studies.

In a different multicenter, randomized, double-blind, placebo-controlled trial, Ziegler and co-workers examined the effects of ALA on neuropathic deficits and positive sensory symptoms in patients with diabetes with distal symmetric polyneuropathy [76]. One hundred and eighty-one patients with diabetes who received oral ALA or placebo once daily were enrolled: 45 patients received ALA 600 mg, 47 patients received ALA 1200 mg, and 46 patients received ALA 1800 mg. The control group, consisting of 43 patients, received only a placebo. The results of the trial demonstrated that the average Total Symptom Score, stabbing and burning pain, and neuropathy symptoms decreased significantly in all three treatment groups compared to those under control. In particular, it has been demonstrated that ALA (600 mg/day) represents the ideal dosage with the optimal risk–benefit ratio.

ALA’s efficacy in treating burning mouth syndrome, a neuropathy-like condition linked to diabetes, poor glycemic control, and the generation of harmful free radicals, was investigated in a study by Femiano and Scully [77]. Sixty patients with continuous burning mouth syndrome participated in the 2-month double-blind controlled study. Patients in the treated group (n = 30) were given ALA in oral tablets of 200 mg, three times a day (600 mg/day) while patients in the control group (n = 30) were given cellulose starch, in similar tablets, 100 mg/day, three times a day. Compared to placebo, ALA treatment produced a notable improvement in symptoms. After 2 months, most patients showed at least some improvement and at the 1-year follow-up, over 70% of patients still showed improvement. According to the study results, ALA is useful in the treatment of burning mouth syndrome.

A 2-year multicenter randomized double-blind placebo-controlled trial was carried out by Reljanovic et al. [78] to examine the impact of ALA on diabetic polyneuropathy. In this research, patients with symptomatic polyneuropathy who were diagnosed with type 1 or 2 diabetes were randomized into three groups: two groups were treated with (i) 6 × 200 mg of ALA (n = 18; ALA 1200 mg/day) or (ii) 3 × 200 mg of ALA (n = 27; ALA 600 mg/day). The third group (n = 20) served as a control and received the placebo. The severity of diabetic neuropathy was assessed using the Neuropathy Disability Score and electrophysiological parameters in several motor and sensory nerves. According to the study, ALA appears to exert a beneficial effect on several aspects of nerve conduction, particularly on sural sensory nerve conduction velocity. The study results also demonstrated that 2-year treatment with ALA (600 and 1200 mg) was safe and was rated as very good/good in tolerability by almost all patients.

Agathos et al. [79] carried out a study to examine the impact of ALA therapy on the quality of life and neuropathic symptoms of individuals with painful diabetic neuropathy. Seventy-two patients (age 18–75 years) who received oral treatment with ALA (600 mg/day) for 40 days were enrolled in this study. Neuropathy Symptom Score, Subjective Peripheral Neuropathy Screen Questionnaire, and Neuropathic Pain scores all showed statistically significant decreases in neuropathic symptoms. The Sheehan Disability Scale, the Neuropathic Pain Symptom Inventory, and the Brief Pain Inventory were also used to evaluate the effects of the therapy on quality of life and the results demonstrated notable improvements. Additionally, fasting triglyceride levels were lower; however, no appreciable changes in body weight, blood pressure, fasting blood glucose, or other lipid values were found. Although this was an open-label study, without a placebo control group, and as such has some limitations, the study results were able to demonstrate that taking ALA was linked to lower triglycerides, fewer neuropathic symptoms, and a better quality of life.

A randomized double-blinded placebo-controlled study by El Nahas et al. [80] evaluated the effectiveness of oral treatment with ALA for a period of 6 months in patients with symptomatic diabetic peripheral neuropathy. Two hundred patients were randomized to receive 600 mg of ALA (treated group; n = 100) or placebo (control group; n = 100) twice daily. Different parameters were used to evaluate the treatment results, such as vibration perception threshold, neurological symptom and disability scores, and visual analogue scale (VAS) for pain, and patients treated with ALA achieved significantly better results than the placebo group. The study demonstrated that oral supplementation of 600 mg of ALA twice daily is an adequate, safe, and effective approach.

### 7.2. Alpha-Lipoic Acid for Treatment of Cardiovascular Disorders

Oxidative stress is responsible for the development of many cardiovascular diseases, such as hypertension, ischemia, heart failure, and atherosclerosis. One of the main factors leading to the development of these diseases is the overproduction of ROS, together with the reduced bioavailability of nitric oxide (NO).

High blood pressure is a major contributing factor to stroke, heart attack, and arterial aneurysms. Since NO regulates vessel wall elasticity and ALA enhances endothelial NO synthesis [6], the role of ALA in the control of high blood pressure has been extensively studied. The capacity of ALA to elevate tissue GSH levels and halt harmful sulfhydryl group modification in Ca^2+^ channels provided the basis for its therapeutic application against hypertension.

#### 7.2.1. Preclinical Studies in Models of Cardiovascular Disorders

In vivo studies on hypertensive rats demonstrated that the administration of ALA induces normalization of systolic blood pressure (SBP) [81,82]. Normalization of aortic superoxide generation and blood pressure have been linked to the restoration of GSH peroxidase activity observed in ALA-fed rats [83]. ALA has also been suggested to inhibit renal and vascular overproduction of endothelin-1, a vasoconstrictor produced by the endothelium [84]. Administration of ALA (in combination with acetyl-L-carnitine) is able to reduce SBP in patients with hypertension and in subjects with metabolic syndrome [6].

Additional in vivo studies (diabetic and control rats) studied the effects of an evening primrose oil (EPO) supplement and ALA supplement on various lipid and hemostatic parameters. The results obtained demonstrated that both ALA and EPO improve blood flow and nerve function. In particular, the marked effects of ALA on lipid and hemostatic risk factors suggest potential antithrombotic and anti-atherosclerotic effects [85].

#### 7.2.2. Clinical Studies About Cardiovascular Disorders

A randomized, double-blind, placebo-controlled clinical trial has been carried out to examine the possible effects of ALA consumption on some cardiovascular risk factors in patients with stroke [86]. Stroke represents a leading cause of death worldwide and is responsible for long-term disability with high personal and social costs in adults. In this study, participants were randomly assigned into two quantitatively equal groups. Subjects in the ALA group took ALA (600 mg/day) for 12 weeks, while patients in the placebo group took a similar placebo capsule (containing wheat flour) every day for 12 weeks. Fasting insulin, fasting blood glucose (FBS), and SBP and diastolic blood pressure (DBP) were measured before and after ALA treatment. Before treatment, the two groups had no statistically significant differences in age, weight, height, body mass index, waist circumference, blood pressure, energy and macronutrient intake, and FBS. After the treatment period, however, SBP (*p* < 0.001), DBP (*p* < 0.001), and FBS (*p* < 0.001) were significantly reduced in the ALA group compared to the placebo group. No significant changes in insulin levels were observed. The results of this study demonstrated that a 12-week supplementation with ALA (600 mg/day) has beneficial effects on both blood pressure and FBS.

ALA supplementation’s effects on adult blood pressure were also examined using a dose–response meta-analysis of randomized controlled trials and a GRADE (Grading of Recommendations, Assessment, Development, and Evaluation)-assessed systematic review [87]. The findings of this review confirm the positive effect of ALA administration on lowering SBP and DBP levels when compared to the control group. Sensitivity analysis reveals that the overall effect sizes calculated for SBP and DBP did not vary significantly after removing each individual study, implying that the study results are reliable, robust, and not dependent on a single study. Improving blood pressure has a substantial impact on mitigating the burden of cardiovascular disease resulting in important public health benefits.

Endothelial dysfunction, an early sign of systemic atherosclerosis, represents a therapeutic target to prevent long-term cardiovascular consequences. A double-blind, placebo-controlled randomized trial has been carried out to evaluate the effect of ALA on endothelial function [88]. Sixty-four young overweight/obese subjects were randomly assigned to oral supplementation with ALA (800 mg/day; n = 32) or placebo (n = 32) and twenty-two normal-weight metabolically healthy children served as controls. Endothelial function was assessed by flow-mediated dilation of the brachial artery and clinical and metabolic risk factors. After treatment for 3 months with ALA, the basal and peak diameter of the brachial artery was significantly increased in treated patients compared to placebo, demonstrating that ALA supplementation, by improving vascular tone, has a beneficial effect on cardiovascular health in overweight/obese young people.

A study was carried out on 40 diabetic patients with Stage I hypertension to determine the effect of a combination of ALA with angiotensin-converting enzyme inhibitors (ACEI) on blood pressure, endothelial function, and proteinuria [89]. Patients were administered ACEI (Quinapril, 40 mg/day) for 8 weeks or ACEI + ALA (600 mg/day) for 8 weeks. At the end of therapy, a change in metabolic parameters was observed in both study groups. Compared to baseline, 24 h urinary albumin decreased by 30% in the ACEI group and by 53% in the ACEI + ALA group. Furthermore, when compared to baseline, flow-mediated dilation increased by 58% in the ACEI group and by 116% in the ACEI + ALA group. SBP and DBP were reduced by 10% with both treatments. The results obtained demonstrated that in diabetic patients with hypertension, ALA enhances the effect of ACEI by reducing proteinuria and improving endothelial function.

### 7.3. Beneficial Effects of Alpha-Lipoic Acid in Patients with Cancer

Cancer is mostly a consequence of somatic mutations in the genes that control cell growth and division. Because ALA can regulate gene expression, it has been hypothesized that it may turn off genes that stimulate the development of cancer.

#### 7.3.1. Preclinical In Vitro Studies in Cancer Models

Several experimental studies in in vitro cancer models have demonstrated that ALA could exert beneficial effects through different mechanisms of action.

As a matter of fact, ALA is able to activate pyruvate dehydrogenase in different cancer cells, inhibiting their proliferation, increasing their apoptosis, and hindering tumor growth [61,90].

Moreover, ALA is able to increase the abundance of cadherin in thyroid cancer cells and inhibit the molecules involved in tumor progression (activated β-catenin and vimentin). ALA pretreatment of breast cancer cell lines increases cell sensitivity to ionizing radiation and blocks transforming growth factor beta signaling inhibiting the migration and invasion of metastatic breast cancer cells [91]. ALA is also able to act on tumor metabolism in in vitro models. Tumor cells convert glucose to lactate and produce ATP through aerobic glycolysis (Warburg effect). Persistent stimulation of aerobic glycolysis in these cells results in the loss of tumor suppressors and disease progression. ALA acts as a cofactor for pyruvate dehydrogenase (PDH), activating it and inhibiting pyruvate dehydrogenase kinase. PDH converts pyruvate to acetyl-CoA, thus preventing lactate formation and tumor cell proliferation [90].

#### 7.3.2. ALA to Improve the Quality of Life of Patients with Cancer: Clinical Studies

A prospective randomized double-blind placebo-controlled study was carried out on patients with breast cancer to investigate the effect of ALA supplementation to counteract the toxicities induced by paclitaxel and doxorubicin [91]. In this study, 64 women with stage II and III breast cancer were divided into two groups: the control group (n  =  32) received 4 cycles of doxorubicin plus cyclophosphamide (every 21 days) followed by weekly doses of paclitaxel for 12 weeks plus placebo tablets once daily. The ALA group (n  =  32) received the same chemotherapy regimen plus ALA (600 mg/day) for 6 months. The results obtained demonstrated that the use of ALA (600 mg/day) as an additional therapy to doxorubicin and paclitaxel induces a significant improvement in the peripheral sensory neuropathy grading (according to National Cancer Institute Common Terminology Criteria for Adverse Events, version 4.0) and in the FACT/GOG-Ntx-12 (12-item neurotoxicity questionnaire) total score. Furthermore, ALA intake induced a significant decrease in serum levels of brain natriuretic peptide, inflammatory marker (tumor necrosis factor-alpha), oxidative stress marker (malondialdehyde), and neurotensin. This study demonstrated that ALA can provide supportive therapy in cancer and may represent a promising adjuvant therapy to attenuate paclitaxel-induced peripheral neuropathy and counteract doxorubicin-induced cardiotoxicity.

### 7.4. Effects of Alpha-Lipoic Acid on Hearing Loss

Hearing loss can be caused by several environmental risk factors, and cochlear redox imbalance is the main mechanism of damage involved in the pathogenesis of noise-induced hearing loss. Based on this observation, several studies have been carried out to evaluate the effectiveness of exogenous antioxidants, including ALA, to prevent or attenuate noise-induced damage.

#### 7.4.1. Preclinical Studies in Ototoxicity Models

A study carried out both in cultured cells and in an animal model (male and female mice) demonstrated the strong beneficial potential of ALA in rescuing ototoxic hearing loss caused by cisplatin. The results of this study showed that ALA can act as a GSH disulfide-reducing agent to increase GSH levels on behalf of GSH reductase. These results obtained both in vitro and in vivo suggest the clinical value of ALA for the therapy of cisplatin-induced ototoxicity [92].

#### 7.4.2. Clinical Studies in Subjects with Hearing Disconmforts

Noise-induced hearing loss (NHIL) is a major cause of hearing loss in developed countries. The cellular basis of NIHL has opened up new avenues for protection through prophylactic compounds such as antioxidants. ALA is a powerful free radical scavenger and an important cofactor for mitochondrial enzymes. A study by Quaranta et al. [93] examined the impact of ALA (BETTERAL^®^) supplementation on NIHL. The purpose of this study was to see how ALA affected the temporary threshold shift (TTS2) caused by a 3 kHz tone in young, normally hearing subjects. In this study, 30 young volunteers with normal hearing were randomly divided into three groups (10 subjects/group). Group A subjects were exposed to a 90 dB HL 3 kHz pure tone for 10 min; Group B subjects were exposed to a 90 dB HL 3 kHz pure tone for 1 h after oral ingestion of 600 mg of ALA; Group C subjects were exposed to a 90 dB HL 3 kHz pure tone after 10 days of oral ingestion of 600 mg of ALA. The results demonstrated that the TTS2 induced by a 90 dB HL 3 kHz tone was not protected in any way by a brief course of ALA. On the other hand, substantial protection at 6 kHz was linked to a 10-day therapeutic dosage supplementation of ALA, indicating its potential to prevent noise-induced hearing loss.

### 7.5. Alpha-Lipoic Acid and Reproductive System

Infertility affects over 15% of married couples worldwide. ALA represents a very promising molecule for infertility, and several studies carried out in animal models have highlighted the influence of ALA on reproductive function, such as pregnancy, oocyte, and sperm cells. Excessive ROS generation by leukocytes in semen and defective sperm can contribute to infertility, and it has been observed that high amounts of ROS render sperm immobile [94]. Therefore, ALA, by reducing the production of ROS, helps to preserve the vitality and motility of the spermatozoa. As a matter of fact, the efficacy of ALA in enhancing the quality and preserving the function of sperm by reducing lipid peroxidation and improving the activity of antioxidant enzymes has been described [95].

ALA can also enhance the developmental potential of oocytes in the mouse by reducing the level of ROS [96].

#### 7.5.1. Preclinical Studies in Experimental Models of Reproductive Function

It has been observed in in vivo experimental models that ALA protects tubal and uterine epithelial cells from the harmful effects (oxidative stress) of cigarette smoke [97]. Moreover, ALA exerts a protective effect on the progression of endometriosis [98].

#### 7.5.2. Alpha-Lipoic Acid and Female Pathologies: Clinical Studies

Concerning gynecological pain, in a review published in 2014, Costantino et al. [99] examined the efficacy of ALA supplements in alleviating neuropathic pain, a frequent and disabling disease that pregnant women may experience due to physical changes and compression during pregnancy and childbirth. Pregnant women have few alternatives because many of the common therapies for neuropathic pain are inappropriate during pregnancy. In order to address this issue, a review was carried out to assess the safety and effectiveness of supplementing with ALA (BETTERAL^®^) to treat neuropathic pain during pregnancy. According to the review, ALA has a number of biochemical functions and actions, including significant anti-inflammatory and antioxidant effects as well as a notable improvement in pain and paresthesia in patients suffering from a variety of neuropathic pain disorders, including diabetic neuropathy, CTS, and low back pain. Furthermore, ALA has a strong safety profile, rendering it a propitious option for the management of many medical conditions. These results suggest that using ALA as a dietary supplement may be helpful in reducing neuropathic pain during pregnancy.

During the first trimester of pregnancy, the risk of miscarriage is high. The effectiveness of ALA treatment in preventing miscarriage has been studied through several clinical trials.

Costantino et al. [100] also studied the effect of treatment with vaginal ALA or progesterone on the resorption of subchorionic hematoma, which may increase the risk of early miscarriage [101] in 62 pregnant women with threat of miscarriage. Patients received progesterone (400 mg/day) in the form of a soft vaginal gel or ALA (10 mg/day) in the form of a vaginal capsule, whereas a control group had no treatment. The treatment lasted 60 days and the development of the subchorionic hematoma was monitored by vaginal ultrasound after 20 days and at the end of the treatment. Although no changes in pelvic pain and vaginal bleeding values were observed in any of the groups, significant improvements and fewer miscarriages due to subchorionic hematoma reabsorption were recorded in the ALA-treated group compared to the progesterone-treated group (*p* < 0.05), suggesting that ALA may be effective for treating patients with threatened miscarriage.

Grandi et al. [102] investigated the anti-inflammatory activities of ALA on cervical inflammation and shortening after primary tocolysis through a pilot, randomized, placebo-controlled study. Thirty-two women with a gestational age between 24 and 30 weeks and hospitalized for a first episode of preterm labor were enrolled. These women were randomly divided into two groups: one group (n = 17) received ALA (400 mg/day) in the form of hard vaginal tablets and the control group (n = 15) received the placebo. The administration lasted 30 days. The analysis of cervicovaginal fluids highlighted a notable increase in the levels of IL-4 and IL-10 in women supplemented with ALA compared to placebo, while no significant changes in the levels of proinflammatory cytokines were observed between the groups. Furthermore, tracking the cervical length using transvaginal ultrasound before and after treatment highlighted that ALA is able to effectively limit the shortening of the cervix compared to the control group.

All these results, although preliminary, encourage carrying out larger randomized controlled clinical trials.

#### 7.5.3. Alpha-Lipoic Acid and Male Pathologies: Clinical Studies

Hodeeb and collaborators [103] evaluated the efficacy of ALA administration in primary infertile males with idiopathic asthenozoospermia. Eighty patients were enrolled and randomly divided into two groups. The treated group (n = 40) received two daily doses of 300 mg of ALA (600 mg/day) and the control group (n = 40) received two daily doses of placebo.

The results of the study demonstrated that ALA supplementation significantly improves the total motility and progressive motility of spermatozoa and the average percentage of sperm viability (*p* < 0.001). In the treated group, a statistically significant increase in semen volume and sperm concentration and a decrease in abnormal morphology were also observed (*p* < 0.001).

A study was carried out to verify whether ALA integration could improve the motility of the sperm in individuals with varicocele undergoing varicocelectomy [104]. Sixty men (aged between 19 and 45 years) with uni/bilateral grade II–III varicocele were enrolled. After undergoing microsurgery varicocele repair, participants were randomly assigned to a treated group (n = 30) or control (n = 30) group. Patients were treated for 80 days with ALA (600 mg/day, treatment group) or with a placebo (control group). Sperm samples were collected before surgery and after treatment. It was observed that in the treated group, the total motility and progressive motility of the spermatozoa were significantly higher than in the placebo group, demonstrating that an 80-day cycle with ALA after surgical repair is able to improve the total and progressive motility of the spermatozoa in subjects affected by varicocele.

In a randomized, blinded, placebo-controlled clinical trial, the effects of ALA supplementation on spermatogram and seminal oxidative stress biomarkers in infertile men were studied [105]. Forty-eight men with idiopathic asthenozoospermia were divided into two groups: the treated group (n = 24) received ALA (600 mg/day) whereas the control group (n = 24) received a placebo. After 12 weeks of treatment, sperm analysis highlighted that the total number of spermatozoa, their concentration, and their motility were significantly higher in the treated group compared to the control group (*p* < 0.001). Furthermore, treatment with ALA also led to a significant improvement in seminal levels of total antioxidant capacity compared to placebo, demonstrating an improvement in the quality of seminal parameters after ALA treatment.

### 7.6. Other Alpha-Lipoic Acid Applications

#### 7.6.1. Alpha-Lipoic Acid Management of Idiopathic Pain

Management of chronic pain with unknown etiology represents a major challenge for physicians as they can only relieve pain with the use of symptomatic drugs which, in some cases, can have significant side effects.

As extensively documented and described previously, ALA has proven to be effective in relieving pain caused by diabetic neuropathy. Based on this evidence, a study was carried out to evaluate the safety and effectiveness of ALA in reducing chronic pain in normoglycemic subjects. The study enrolled 210 normoglycemic adults (aged 18–75 and of either sex) suffering from chronic pain. In particular, 57 subjects were analyzed with primary neuropathic pain, 141 subjects with arthralgia of unknown etiology, and 12 subjects with idiopathic pain myalgia [106]. The subjects enrolled were divided into three groups: subjects in Group 1 (n = 70) received two 400 mg ALA tablets (800 mg/day), those in Group 2 (n = 70) received one 400 mg ALA tablet (400 mg/day), while patients in Group 3 (n = 70) received placebo. Patients underwent two visits (at baseline = t0 and after 2 months = t1). Results of this study demonstrated that ALA supplementation significantly reduces the intensity of pain in a dose-dependent manner, as measured by the two most commonly used unidimensional pain intensity scales, the Pain Numeric Rating Scale (NRS) and the VAS. Based on these observations, ALA was effective regardless of the type of pain (arthralgia, neuropathic pain, and myalgia with unknown etiology) and could represent a feasible option compared to commonly used analgesic drugs, because of its safety and efficacy.

#### 7.6.2. Alpha-Lipoic Acid as an Anti-Aging Compound

Globally, the average age of the population has risen over time. Skin sagging, wrinkles, and cutis laxa are all symptoms of aging [107]. Aged skin has been linked to reduced barrier function, dryness, and a higher risk of skin illness [108]. To delay skin aging and reduce the negative consequences of aging in general, a strategy for the generation of anti-aging compounds is needed. Both endogenous and exogenous factors can accelerate skin aging [109]. Intrinsic skin aging is linked to reduced cell replication capacity and the weakening of the extracellular matrix. Keratinocytes and fibroblasts are the main producers of ROS that play a crucial role in endogenous skin aging [109]. Environmental oxidative agents are the root cause of exogenous aging, and skin aging triggered by extrinsic factors varies from other tissues’ aging [109,110]. Therefore, an antioxidant action is necessary in order to prevent the oxidative action that occurs in the tissues. As described above, in nature, ALA exists in two forms, as a cyclic disulfide (oxidized form) or as an open chain with the name of dihydrolipoic acid, which are easily interconvertible [8]. ALA is a powerful antioxidant because it is able to neutralize ROS and reactive nitrogen species (RNS) [111]. The chelation of metals inhibits the free radical formation and the oxidative damage [112]. For its properties, ALA is used as a functional ingredient in the preparation of cosmetic products that aim to achieve anti-aging, antioxidant, and repair effects of damages caused by the sun’s rays. ALA in anti-wrinkle creams is a safe ingredient if utilized at normal concentrations of use, which are between 0.1 and 0.05%. When applied to the skin, it is incorporated into the inner layers and offers antioxidant protection against the harmful UV radiation of the sun, and a randomized, placebo-controlled, double-blind study in 33 women (mean age 54.4 years) demonstrated that 12 weeks of treatment with a cream containing 5% ALA improved photoaging-related clinical features of facial skin. [113]. In addition, ALA regenerates the levels of other antioxidants, such as GSH, which help protect against skin damage and can reduce the signs of aging. In fact, it has been shown that applying a cream containing ALA to the skin reduces wrinkles, lines, and skin roughness without side effects [114,115]. In a mouse model, it has also been demonstrated that ALA has a beneficial effect on skin damage caused by cigarette smoke [116].

#### 7.6.3. Effect of Alpha-Lipoic Acid on Muscle Damages

A double-blind, randomized, controlled study with a cross-over design was conducted to test the effects of ALA on the recovery of strength and muscle performance of athletes undergoing intense physical training [117]. Seventeen athletes were enrolled and divided into two groups (ALA and placebo). The intervention phase took place twice (ALA supplementation or placebo) and was divided into two parts. In the first part, the effect of a single application of ALA (150 mg) after intense training was examined. After a 72 h recovery, the second part of the exam was performed through a short-term chronic ALA supplementation and a 6-day training protocol. During the 6 days, subjects received ALA or a placebo 2 h before and immediately after the training session (a total of 300 mg of ALA). At specific times (T0, 3 h, 24 h, and 7 days), blood samples were taken and markers of muscle damage, inflammation, and oxidative stress were studied. The results showed that the administration of ALA during periods of intensive training resulted in reduced muscle damage and inflammation together with increased recovery.

Another study evaluated the antioxidant action of ALA in trained men exposed to muscle-damaging exercises [118]. In particular, the indices of the GSH antioxidant system and oxidative damage were compared between trained subjects and resistance-trained subjects. About 33 men were enrolled, 13 trained and 20 untrained, randomly assigned to the ALA-treated group (600 mg/day, for 8 days) and the placebo control group. All subjects performed an isometric/isokinetic effort of the quadriceps muscles. The study demonstrated that oral administration of ALA reduces oxidative injury through modulation of the pro-antioxidant response in trained men performing muscle-damaging exercises by significantly increasing the erythrocyte levels of GSH, glutathione reductase (GR), and glutathione peroxidase (GPx).

#### 7.6.4. Alpha-Lipoic Acid and Viral Infections

Since pharmacological approaches aimed at counteracting virus-induced oxidative stress are known to be useful for the treatment of viral infections and ALA is a potent antioxidant, several studies have been conducted to evaluate its antiviral effects [119]. Results from these in vitro and in vivo studies have demonstrated that ALA can act on the course of viral infections by modulating biochemical, virological, immunological, and neurological parameters associated with these pathologies.

A randomized, double-blind, placebo-controlled trial was carried out to evaluate whether ALA supplementation can improve lymphocyte function in HIV-infected subjects with a history of failure to respond to highly active antiretroviral treatment [120]. An eye clinic at a hospital in San Jose County and a research clinic in San Francisco, California participated in the study. Thirty-three HIV-infected subjects of both sexes and aged between 44 and 47 years were enrolled. Patients in the treated group received 300 mg of ALA three times daily (900 mg/day) while those in the control group received a placebo. The treatment lasted 6 months, and the results obtained showed that in the subjects treated with ALA, the mean level of total GSH in the blood and CD4+ T-cell proliferation were significantly elevated compared to the subjects taking the placebo. The trial demonstrated that ALA supplementation exerts a positive effect on patients with acquired immunodeficiency syndrome (AIDS).

An increasing body of literature data indicates that ALA may boost also human host defense against SARS-CoV-2 [119]. It is known that the severity of COVID-19 is directly related to the massive release of proinflammatory cytokines. This “cytokine storm” promotes a self-sustained inflammatory reaction which, contributing to the destruction of the endothelium, leads to “acute respiratory distress syndrome”, multiple organ failure, and death. As already reported, ALA is able to inhibit NF-kB signaling, thereby decreasing the secretion of proinflammatory cytokines and restoring nitric oxide synthase activity by improving endothelial function [121]. Therefore, ALA can prevent severe forms of COVID-19 by decreasing the secretion of proinflammatory cytokines,.

A randomized, single-blind trial was performed by Zhong and colleagues [122] to evaluate the clinical efficacy and safety of ALA in 17 severely ill COVID-19 patients (mean age 63 years) admitted to JinYinTan Hospital in Wuhan. Patients in the treatment group (n = 8) were administered intravenous ALA (1200 mg/day) for 7 days in addition to standard medical care, while patients in the control group (n = 9) were treated with saline infusion in addition to standard medical care for 7 days. Although the sample size was too small to draw solid conclusions, a positive response was recorded in subjects treated with ALA. In addition, the authors found a slow increase in the Sequential Organ Failure Assessment (SOFA) score.

These preliminary results suggest that ALA might contribute to counteract viral infections. In particular, the biological activities and properties of ALA make it a potential candidate for the treatment of SARS-CoV-2 infection. However, further randomized control studies are needed to evaluate and confirm the efficacy of ALA as a potential adjuvant therapy in COVID-19 infection.

#### 7.6.5. Alpha-Lipoic Acid for Management of Neurodegenerative Disorders

The incidence of neurodegenerative disorders has significantly increased worldwide due to the increase in life expectancy.

Alzheimer’s disease (AD) is a neurodegenerative disease that develops with aging and affects behavioral and cognitive functions. AD is mainly initiated due to the imbalance between the formation and clearance of amyloid-β (Aβ) and is characterized by an accumulation of abnormal neuritic plaques and neurofibrillary tangles of hyperphosphorylated tau. Metal ions, being toxic to the nervous system and causing oxidative stress, have been reported to participate in the pathogenesis of AD [123]. As previously described, ALA is a water- and fat-soluble antioxidant capable of crossing the blood–brain barrier to reach the central nervous system. It has been demonstrated that ALA is able to protect hippocampal neurons in primary culture from both Aβ peptides and Fe/H_2_O_2_ mediated toxicity and that its metal chelating properties may increase the extraction of Aβ peptides from cortical areas [124]. Moreover, in an in vivo model of AD (mouse model), ALA has been shown to enhance the extraction of Aβ from the frontal cortex [125]. The development of AD has been linked with mitochondrial oxidative stress in microglia. Aβ interacts with microglial receptors activating downstream pathways that cause mitochondrial damage and exacerbate inflammation and cytotoxicity. In particular, an increase in ROS generation, a decrease in energy storage, and a loss of mitochondrial membrane potential have been observed [126]. ALA supplementation might be useful in AD development because it can improve mitochondrial function in patients with AD by acting as a cofactor of the mitochondrial enzyme complex and antioxidant [127]. Hager and collaborators carried out two studies on the effect of ALA administration in patients with probable AD and related dementias. In a first study [128], nine patients (eight males and one female) receiving choline esterase inhibitors were treated with ALA (600 mg/day) for about 1 year. It was observed that treatment with ALA led to a stabilization of cognitive functions demonstrated by constant scores in two neuropsychological tests (the Mini-Mental State Examination and the cognitive subscale of the AD assessment score). Forty-three patients with mild-to-moderate dementia were enrolled in the second study. Before being treated with ALA (600 mg/day), patients received a standard 3-month course of treatment with a choline esterase inhibitor [129]. The study lasted 2 years. The results obtained demonstrated that the progression of the disease in patients treated with ALA is significantly lower than that in untreated patients or those treated with choline esterase inhibitors. Although both these studies are very preliminary, the data obtained suggested that ALA treatment could be a successful “neuroprotective” treatment option for AD and related dementias.

Multiple sclerosis is an autoimmune disease of the central nervous system characterized by neurodegeneration and demyelination. Relapsing-remitting multiple sclerosis, with alternating relapses and remissions, commonly represents the initial course. After approximately 20 years, most of these patients will develop secondary progressive multiple sclerosis characterized by progressive neurodegeneration without defined periods of remission. The effect of ALA on this secondary progressive multiple sclerosis was studied in a randomized, double-blind, placebo-controlled phase II clinical trial [130]. Fifty-one patients (ages 40–70 years) were enrolled: 27 patients received ALA (1200 mg/day) and 24 patients received a placebo. After 2 years of treatment, the results obtained demonstrated a 68% reduction in the rates of brain atrophy in patients treated with ALA compared to placebo.

## 8. Combinations of ALA and Other Compounds: Clinical Studies

The generation of free radicals plays a central role in the development of various pathological conditions, mainly of multifactorial origin. This observation led to the development of research, in which the use of a combination of ALA with other compounds, mainly with other antioxidants, was evaluated.

### 8.1. Clinical Studies on the Ability of Combinations of ALA and Other Compounds to Repair Nerve Damage

A study by Bertolotto and Massone [131] evaluated the efficacy of a combination of racemic ALA (BETTERAL^®^), SOD, vitamin E, and selenium in the treatment of diabetic neuropathy. Fifty patients with both motor and sensory nerve conduction impairments and diabetes mellitus participated in the ambulatory care settings trial. Electroneutrophic markers and pain perception significantly improved after 4 months of combined ALA and SOD treatment (*p* < 0.001). According to the study, the combination of two powerful antioxidants, like ALA and SOD, produced considerable improvements in both subjective and objective metrics. The sensory nerve conduction showed the greatest recovery. The results of this study show promising data for non-invasive treatment of diabetic neuropathy, providing new possibilities for further research.

Di Geronimo et al. [132] examined the effects of racemic ALA (BETTERAL^®^) supplementation in people with CTS. This syndrome is a common peripheral mononeuropathy that severely reduces the quality of life and the usual activities of people who are affected. Pharmacological therapy aimed at reducing and rectifying nerve damage may be used to control the disease in its initial stages, while surgery is only performed in severe cases. The study was carried out in 112 subjects with moderately severe CTS, comparing the effectiveness of a multivitamin B preparation (Vit B6 150 mg, Vit B1 100 mg, Vit B12 500 μg daily) for 90 days with a fixed association of ALA (600 mg/day) and gamma-linolenic acid (GLA, 360 mg/day). Compared to the multivitamin group, the ALA and GLA association was more successful in lowering symptom scores and functional impairment as determined by the Boston CTS Questionnaire (BCTSQ), which gives detailed information about symptoms and symptom severity when executing specific actions. In addition, unlike ALA/GLA, the multivitamin product did not show a statistically significant improvement in electromyography. Finally, ALA/GLA demonstrated notable efficacy in reducing symptoms and functional deterioration measured by the Hi-Ob scale (a historical-objective scale based on clinical history and physical examination), while the multivitamin group showed a less marked improvement. The results of this study demonstrate that the fixed combination of ALA and GLA, in particular, in the early stages of the disease, represents a useful tool for managing symptoms and improving the course of CTS. Notably, these results agree with a recent review confirming the safety and effectiveness of the ALA and GLA combination for improving DPN [133].

The clinical utility of oral supplementation with a product (in tablet form) containing ALA (300 mg), curcumin phytosome (a form of curcumin coated with phospholipids, 500 mg), and B vitamins (vitamin B1, 1.05 mg; vitamin B2, 1.2 mg; vitamin B5, 4.5 mg; vitamin B6, 1.5 mg) was studied in 180 patients (44 males and 136 females, mean age: 57.9 ± 14.8 years) with CTS, scheduled to undergo surgical decompression of the median nerve [134]. Patients were divided into three groups: patients in Group A (n = 60, control) received no treatment; Group B patients (n = 60) received oral supplementation twice daily for 3 months before and 3 months after surgery; Group C patients (n = 60) received oral supplementation twice daily for 3 months before surgery. Patients in Group B (treatment for 6 months) showed significantly lower nocturnal symptom scores than subjects in Group A at both 40 days and 3 months after surgery (both *p* < 0.05). Furthermore, Group B patients had significantly fewer positive Phalen tests at 3 months than the other study groups (*p* < 0.05) demonstrating that oral supplementation with ALA, curcumin phytosome, and B vitamins two times a day both before and after surgery is safe and effective in patients with CTS scheduled to undergo surgical decompression of the median nerve. Since no significant improvements were observed in Group C patients, the results indicate the importance of maintaining supplementation after surgery.

Luchetti and collaborators [135] carried out a multicentric observational study on 377 patients suffering from CTS to evaluate the management practices of this pathology in Italy. A wide range of interventions were prescribed, such as pharmacological, physical, and neurotrophic therapies. Neurotrophic formulations containing ALA (BETTERAL^®^) were used to treat a subgroup of 303 patients. At the end of the follow-up, a significant improvement was observed in both functional deterioration and overall symptoms. In particular, a significant decrease in BCTSQ and in NRS (an 11-point unidimensional scale that assesses pain intensity in adults) was observed (both *p* < 0.001).

### 8.2. Clinical Studies on the Ability of Combinations of ALA and Other Compounds to Relieve Back Pain and Neck Pain

A study has been carried out in order to better understand how a combination of ALA (BETTERAL^®^), SOD, vitamin E, and selenium affects functional activity, analgesic use, and pain perception in patients with chronic lower back pain (LBP) [136]. This prospective non-randomized, open-label study involved 98 adult subjects without inflammatory or neoplastic diseases and with chronic LBP lasting at least 6 weeks and was conducted at the TAMMEF (Therapeutic Application of Musically Modulated Electromagnetic Fields) outpatient center of the University of Siena. Patients received treatment with ALA (600 mg/day) and SOD (140 IU/day) for 60 days. The Roland Morris Disability Questionnaire (RMDQ) and Pain Rating Scale (PRS) were utilized in the trial to document any adverse events and changes in pain perception and functional activities. Results of this study showed that the use of analgesics was significantly reduced after treatment, as demonstrated by the fact that, compared to 73.5% at baseline, only 8% of patients continued to take them after treatment (*p* < 0.01). Furthermore, after 40 days of therapy, there was a statistically significant improvement in functional impairments and subjective pain. Overall, the study found that subjects with chronic LBP who received oral treatment with ALA and SOD had increased functioning and used fewer analgesics.

A prospective, randomized, open study on ALA (BETTERAL^®^) supplementation for patients with chronic neck pain (CNP) was published by Letizia Mauro et al. [137]. In particular, it has been examined whether a combination of ALA and SOD could enhance pain management and the effectiveness of physiotherapy (PT) in patients with CNP. A total of 96 outpatients were enrolled in the trial and randomized to receive 60 days of PT and ALA (600 mg/day) plus SOD (140 IU/day) (Group 1; n = 51) or only PT (Group 2; n = 45). A modified Neck Pain Questionnaire (mNPQ) and a VAS were used to measure pain. After 1 month, there was a significant decrease in the VAS and mNPQ scores for both groups. Nevertheless, Group 1 displayed a further decline in VAS (*p* < 0.001) at 60 days, but Group 2 did not show any progress at all. Furthermore, at 60 days, the mNPQ revealed a significant improvement in neck pain for a larger number of patients in Group 1 compared to Group 2 (*p* < 0.01). Additionally, Group 1 patients demonstrated higher compliance with the recommended PT (*p* < 0.05). Given that ALA and SOD are antioxidants effective against both nerve inflammation and disease evolution, these preliminary results point to the possibility that using them in conjunction with PT may be a helpful treatment for CNP.

### 8.3. Clinical Studies on the Ability of Combinations of ALA and Other Compounds to Relieve Neuropathic Pain

A multicentric observational prospective study was conducted by Checchia et al. [138] with the aim of giving detailed information on the clinical manifestation of sciatic neuropathy and how it is managed in a real-world setting, with an emphasis on the outcomes of a multimodal approach that combines pharmacological and physical therapy. The study has been carried out in 44 specialized tertiary Italian centers for physical medicine and rehabilitation, orthopedics, neurology, neurosurgery, and rheumatology. A specific medical record containing information on diagnosis, treatment, and outcomes was used to create a shared management plan for lower back pain (LPB) with sciatica. Validated questionnaires were used to assess pain, disability, and quality of life both at baseline and during a 2-month follow-up. Three hundred and ninety-four patients with sciatica and chronic LPB (mean age 55.7 ± 14.1 years, 57.1% female) who showed some difference in their clinical presentation were included in the study. Patients were given a variety of therapeutic options at baseline, such as medications, physical therapy, and neurotrophic treatments. A combination of neurotrophic agents containing ALA (BETTERAL^®^) was administered to a subgroup of 312 patients. A 2-month follow-up revealed a significant (*p* < 0.001) improvement in functional disabilities and perceived pain, as measured by the Brief Pain Inventory (BPI), (RMDQ), and NRS. The study findings point to the potential benefits of a multimodal strategy that combines pharmaceutical, physical, and ALA therapy for the treatment of LBP associated with sciatica.

A study on the effects of ALA (BETTERAL^®^) supplementation on patients with radicular neuropathy was published in 2009 by a research group under the direction of Dr. Ranieri [139]. The purpose of the study was to evaluate the impact of ALA and GLA in addition to physical exercise on neuropathic pain and positive sensory symptoms in patients with compressive radiculopathy syndrome, caused by disc-nerve root conflict. Appropriate prevention and treatment are crucial because these syndromes have the potential to become chronic and disenabling. In this observational cohort trial, 203 patients were enrolled and divided into two groups: one group, consisting of 101 patients, received oral doses of ALA (600 mg/day) and GLA (360 mg/day) together with a 6-week rehabilitation program; the other group, consisting of 102 patients, received the rehabilitation program only. Multiple scales were used to assess patient progress, and primary and secondary outcomes were evaluated at different intervals. The findings demonstrated that neuropathic symptoms and deficits in patients with radicular neuropathy were significantly improved by oral treatment with ALA and GLA in addition to rehabilitation therapy. Furthermore, the ALA and GLA groups demonstrated improvements in quality of life. In summary, this study emphasizes how patients with compressive radiculopathy syndrome may benefit from receiving ALA and GLA in addition to rehabilitation therapy.

### 8.4. Clinical Studies on the Application of Combinations of ALA and Other Compounds in the Treatment of Cardiovascular Diseases

A pilot study published in 2013 by Manolescu et al. [140] evaluated the impact of a nutraceutical supplement containing ALA (BETTERAL^®^), as the primary functional ingredient, plus GLA, B vitamins, vitamin E, and selenium on serum paraoxonase-1 (PON-1) lactonase activity in patients recovering from acute stroke. Twenty-eight post-acute stroke patients were recruited for the study and randomly assigned to either the (+) ALA or (−) ALA study groups—that is, to receive two softgels of ALA daily (corresponding to ALA 600 mg/day) or not. PON-1 lactonase activity (LACTA) has been measured in serum from blood samples collected at the beginning and at the end of treatment. The findings demonstrated a significant increase in PON-1 LACTA in the (+) ALA group over the course of the study (17.6 ± 3.2 vs. 27.6 ± 3.5, *p* = 0.002). According to these results, ALA can induce a physiologically relevant increase in LACTA in post-acute stroke patients, allowing this enzyme to promote redox correction.

This topic was further explored in a 2014 study by the same research team. This study assessed the impact of GLA, B vitamins, vitamin E, and selenium along with ALA (BETTERAL^®^) on the redox status of erythrocytes in 28 patients who had suffered a recent acute stroke [141]. Blood samples were obtained at the start and end of the 2-week trial, during which patients were randomly assigned to receive either active supplementation (2 softgels/day corresponding to ALA 600 mg/day) or no supplementation at all. Results of the study showed that patients in the active group had significantly higher SOD and GSH reductase (GRed) activities, suggesting that the erythrocytes’ redox status had been corrected. On the other hand, the concentration of total thiols did not significantly increase, and catalase and GSH transferase (GT) activities decreased. Only SOD, GT, and GRed activities were found to be influenced by ALA plus GLA consumption, according to multiple regression analysis. To find out if administering ALA over an extended period of time could have a more substantial effect on the antioxidant system of erythrocytes, more research is required. Taken together, the results of this study imply that ALA may be a promising dietary supplement for reversing the pro-oxidant state linked to stroke; however, more research is needed to fully understand its effects.

A study conducted by Cinteza et al. [142] examined the effect of the same nutraceutical supplement containing ALA (BETTERAL^®^), plus GLA, B vitamins, vitamin E, and selenium and the inflammatory state of patients undergoing rehabilitation post-acute stroke (BETTERAL^®^). An inflammatory reaction following a stroke may persist for several months. Furthermore, a pro-inflammatory status is associated with stroke-related comorbidities like diabetes mellitus, dyslipidemia, cardiovascular disease, and atherosclerosis. Twenty-eight people participated in a pilot study that was split into two groups: (+) ALA and (−) ALA. Each group consisted of 14 subjects (seven females and seven males). The two groups adhered to the same rehabilitation regimen, and their respective medication regimens did not differ significantly. On the other hand, for 2 weeks, the (+) ALA group took two softgels per day of ALA, GLA, B vitamins, vitamin E, and selenium (corresponding to ALA 600 mg/day). Blood samples were obtained at the start and end of the research period, and the levels of several inflammatory markers, such as interleukin-1 alpha (IL-1α), interleukin-6 (IL-6), TNF-α, soluble ICAM-1, and myeloperoxidase, were examined. The findings demonstrated that the levels of IL-1α (9.9% ± 3.7, *p* = 0.013) and IL-6 (26.5% ± 8.2, *p* = 0.003) were only significantly reduced in the (+) ALA/GLA group. The ALA/GLA treatment was found to be the cause of the considerable reduction in IL-6 levels (*p* = 0.008) by multiple regression analysis. Moreover, there was a statistically significant difference in the percentage of IL-6 variation between the two groups (8.4% ± 11.5 vs. −26.5% ± 8.2, *p* = 0.034). These results imply that ALA and GLA together may have anti-inflammatory benefits for patients with stroke recovering from an acute episode.

### 8.5. Clinical Studies on the Application of Combinations of ALA and Other Compounds in the Treatment of Oncologic Diseases

Several human studies have been published suggesting an antitumor activity of ALA, usually in combination with other compounds, or examining how ALA affects outcomes in patients with prognosis for various types of cancer.

Encouraging results have been reported in the treatment of pancreatic cancer using intravenous ALA (300 to 600 mg twice weekly) plus low-dose oral naltrexone (4.5 mg once daily) [143]. The results of the study, despite being conducted on only four patients, suggest that this integrative therapy should be considered, given its lack of toxicity and its ability to prolong the life of a patient considered terminal.

Twenty-eight patients (27 males and 1 female) with advanced solid tumors who achieved an objective response or stable disease following chemotherapy were enrolled in an open-label, non-randomized phase II study. Patients received maintenance treatment with the combination of subcutaneous recombinant IL-2 (rIL-2) plus medroxyprogesterone acetate (MPA) plus ALA (300 mg/day, per os) and N-acetyl cysteine (NAC) in an intermittent and repeated schedule over a long period (median duration 10 months; 6–30+) [144]. The results obtained demonstrated that the combination of rIL-2 with MPA, ALA, and NAC had very low toxicity and determined the improvement of the patient’s biological markers. In this study, the 1-year survival rate was 72.2%.

Comparable results were obtained by the same research group in a subsequent study conducted on 42 patients (39 males and 3 females) using the same maintenance treatment [145]. The median duration of treatment was 12 months (range 4–32) and the 1-year survival rate was 73.8%.

The effect of a combination of intravenous ALA (600 mg/day; one patient was unable to receive intravenous ALA and was switched to oral ALA) with hydroxycitrate and low-dose naltrexone (metabolic treatment) was also investigated [143]. This study was carried out and enrolled 10 patients with advanced cancer, who had not responded to standard chemotherapy and with a life expectancy of 2 to 6 months. The results obtained showed that metabolic treatment has very limited toxicity as no patient experienced major side effects. Furthermore, patients lived several months without symptoms. These results suggest that targeting cancer metabolism is an approach to be explored in advanced disease [146].

ALA has also been used as supportive care for patients with cancer. A combination of ALA (240 mg/day), Boswellia serrata (40 mg/day), methylsulfonylmethane (200 mg/day), and bromelain (20 mg/day) has been used in 25 patients (6 males and 19 females) with chemotherapy-induced peripheral neuropathy (CIPN). CIPN was evaluated at the enrollment visit and repeated every 3 weeks for up to 3 months. Patients well tolerated the treatment and a decrease in pain and sensory and motor neuropathic impairment after 3 months of treatment was observed [147].

Thus, preliminary findings indicate that ALA, especially when combined with other substances, may improve quality of life and may even have some antitumor effects; however, randomized controlled trials are still needed.

### 8.6. Effect of Combinations of ALA and Other Compounds on Female Pathologies: Clinical Studies

A recent systematic review investigated the potential benefits of ALA in women with polycystic ovary syndrome (PCOS) and confirmed the improvement of different cardiometabolic factors (such as fasting blood sugar—FBS and HOMA-IR) as well as hormonal parameters [148].

As far as female pathologies are concerned, it has been observed that the administration of ALA and/or myo-inositol (400 and 1 mg/day, respectively) improved the hormonal and metabolic aspects in 90 obese women with PCOS and positively influenced the menstrual rate. In particular, myo-inositol modulated oral glucose tolerance tests (OGTTs) in polycystic ovary syndrome (PCOS) without familial diabetes, ALA increased insulin response to OGTT and metabolic parameters in all patients, and the combination of myo-inositol + ALA improved hormonal and metabolic aspects and insulin response to OGTT in all patients. While myo-inositol was less effective in the presence of familial diabetes, its combination with ALA was effective on all patients with PCOS regardless of familial diabetes [149]. The combination of ALA and D-chiro-inositol also exerts a positive effect on the metabolism of women with PCOS [150,151], while the combination of ALA, NAC, and bromelain is able to significantly reduce pelvic pain associated with endometriosis [152]. Further insights into the use of ALA, in combination with other compounds, in obstetrics and gynecology can be found in the review by Di Tucci et al. [153].

## 9. Conclusions

Based on their remarkable antioxidant properties, both ALA and its reduced form DHLA are considered “universal” antioxidants as they act both in the membrane and in the aqueous phases. ALA and DHLA directly extinguish a variety of reactive oxygen species, inhibit reactive oxygen generators, and spare other antioxidants.

The results of the clinical studies included in this review highlighted that ALA is a molecule capable of preventing or slowing down various conditions linked to the progression of various diseases, such as neurodegenerative diseases, diabetes, and myocardial and cerebral ischemia–reperfusion injury. In particular, since there is a demand for scientifically supported nutraceuticals that maintain nerve health, its effectiveness on nerve discomfort deserves special consideration.

Furthermore, other studies highlighted how the interesting antioxidant properties of ALA together with its interaction with other important antioxidants such as vitamin E, vitamin C, and GSH have important applications that should be further explored.

Taken together, the results of the studies reported in this review have demonstrated the beneficial effects of ALA against various disorders.

However, it is known that a randomized, controlled clinical trial is the prerequisite for evaluating the efficacy of a given treatment. Clinical trials carried out to establish the efficacy of treatment must be conducted on a significant number of subjects using fundamental methodological features such as a credible control condition and random assignment to the experimental and control intervention. In particular, the selection of an appropriate control or comparison group is required to determine, all other things being equal, whether a treatment is efficacious for a given illness, whether it has benefits over other therapies for the illness, and how it can achieve its effects.

The clinical trials conducted have demonstrated that ALA might be considered safe and was well tolerated even at high doses. In addition, it represents an effective therapy in improving the quality of life of patients affected by various disorders. Nevertheless, not all the trials possessed the essential prerequisites to confirm the true potential of this approach.

Therefore, although our review aimed to broaden our knowledge on the clinical applications of ALA, further randomized, double-blind, placebo-controlled clinical trials with adequate sample sizes, homogeneity of population studied, statistical power, as well as longer duration are (still) needed to confirm the clinical benefit of this multifunctional compound and extend its use in routine medical practice.

However, we can conclude that ALA supplementation has proven to be a promising agent for improving the quality of our life and nerve health.
